# Awareness of professional rules among Iranian nurses: a cross-sectional study

**DOI:** 10.1186/s12912-018-0324-9

**Published:** 2018-12-19

**Authors:** Azam Faraji, Amir Aryan, Faranak Jafari, Alireza Khatony

**Affiliations:** 1 Social Development and Health Promotion Research Center, Kermanshah University of Medical Sciences, Kermanshah, Iran; 2 Students Research Committee, Kermanshah University of Medical Sciences, Kermanshah, Iran

**Keywords:** Nurse, Rules, Knowledg

## Abstract

**Background:**

One of the main responsibilities of professional nurses is protecting themselves against legal complications. Hence, they have to be sufficiently aware of the professional rules. This study examines the Iranian nurses’ awareness of professional rules.

**Methods:**

A total of 260 nurses were randomly selected from among the nurses working at various wards and included in this cross-sectional descriptive analytical study. Data were collected using a researcher-made questionnaire. The collected data were then analyzed using descriptive (mean and frequency percentage) and inferential (Kolmogorov-Smirnov, Mann-Whitney U, and Kruskal-Wallis) statistics.

**Results:**

The nurses’ mean awareness of professional rules was 28.3 ± 4.0 out of 37. There was a significant relationship between the mean awareness of the nurses and ward (*p* = 0.001). However, this relationship was not significant regarding demographic variables age, sex, marital status, job title, working experience, education and history of participation in retraining courses on professional rules.

**Conclusion:**

A significant number of nurses were not adequately aware of professional rules, which can put them and their working organization at serious risks. Some measures such as holding web-based or in-person training courses and providing educational booklets and pamphlets can be helpful in this regard.

## Background

All members of a medical team, regardless of how skillful they are, may make mistakes during patient care [1–3]. Meanwhile, nurses are more likely to make errors due to frequent contact with patients [1]. Even in developed countries such as the United States, the number of nursing errors reported by National Practitioner Data Bank in 2011 was twice the figure reported in 2002 [4]. The results of a study indicated the prevalence of medication errors to be 79.2% among nurses [1]. Another study found a prevalence of 19.5 medication error per nurse during three months [5].

Nursing errors are a global and costly issue which may cause harm and even death among patients [6, 7]. These errors are not only problematic for the nurses and but also affect the public image of the nursing profession [6–8]. Today, despite the qualitative and quantitative improvements of the healthcare system, the patients’ complaints about nurses and other members of medical teams are increasing and causing different legal complications [3, 9–11]. The number of nurses summoned to the court is increasing everyday [12]. International reports also indicate an ascending rate of complaints about healthcare providers, including nurses in various countries [11–13]. A survey reported the increase of complaints from 22,500 cases in 2002 to 25,000 cases in 2006 [14]. The rise in the number of complaints can be caused by increased public awareness of Patients’ Bill of Rights, improper interaction between nurses and patients, failure to register nursing measures, mental and physical fatigue due to high working pressure, failure to provide sufficient information to the patients, and lack of awareness of professional rules [6, 11–15].

If the nurses and healthcare centers are found guilty by legal authorities, there is a possibility of being sentenced to pay heavy fine [8]. In Italy, an annual fine of more than 10 million Euros is paid to the patients by hospitals as the compensation for damages due to medication errors [11]. In a case, a nurse was charged to pay a fine of one million and five hundred dollars because of leaving an arterial line in ulnar artery [16].

Undoubtedly, one of the main responsibilities of the professional nurses is to protect themselves against legal complications. Therefore, they have to be sufficiently aware of the professional rules in this regard [6, 7, 11, 13]. However, despite this fact, the results of the previous studies have indicated insufficient awareness among most of the nurses. In this regard, the results of a study showed that most of Indian nurses had poor knowledge about professional rules [4]. Another study conducted in Iran found that about half of the nurses and nursing students lacked knowledge about professional rules [17]. In another study, about 20% of the Barbados nurses had low knowledge about and awareness of professional rules and 34% of them were unaware of the nursing codes 1 [18]. In another study, only 25% of German nurses had sufficient awareness of professional code of ethics [19].

The results of the above-mentioned studies indicate nurses’ insufficient awareness of professional rules. The increasing trend of complaints about nurses and lack of sufficient information about their awareness of professional rules made the researchers to conduct this study. This study aimed to examine the awareness of professional rules among nurses working at hospitals affiliated with Kermanshah University of Medical Sciences (KUMS). The results of the present study are hoped to be an effective step to promote the nursing profession.

## Methods

### Study questions

- How aware are nurses of professional rules?
- What is the relationship between the nurses’ awareness of professional rules and their individual characteristics?

### Design

This was an analytical cross-sectional study.

### Sample

The population of study included all the nurses working at seven educational hospitals affiliated with KUMS in Kermanshah-west of Iran. The samples consisted of 260 undergraduate and graduate nurses in various medical and surgical wards, including emergency, intensive care unit (ICU), critical care unit (CCU), dialysis, internal medicine, obstetrics, pediatrics, general surgery, pediatrics, oncology, psychology, neurology, ENT and eyes. The sample size was proportional to the percentage of nurses employed in each hospital.

The inclusion criteria comprised of having a BSc. or MSc. degree in nursing, having at least 6 six months of work experience in nursing, and consent to participate in the study. The exclusion criteria included not completing the questionnaire. Sampling was done by stratified random sampling, and each hospital formed a stratum. Inside each stratum, random sampling was done using a random number table. For this purpose, the researcher collected the list of nurses working in different parts of each hospital from the nursing office. Samples were selected using the random numbers table.

### Instrument

A researcher-made questionnaire containing two parts was used to collect the data. The first part included 11 items regarding the demographic information of the participants such as age, sex, marital status, education, working experience, job title, passing retraining courses on professional rules, and history of patients’ complaints. The second part included 37 items about legal standards of nursing, various dimensions, including description of nursing job, documentation of nursing reports, various types of nursing failures and misconduct, and Patients’ Bill of Rights.

The questionnaire was developed during several joint sessions with experienced professors in professional ethics and rules as well as senior experts working in the provincial forensics medicine of Kermanshah. Also, the most commonly referred claims against nurses in the forensic centers of Kermanshah province during a 5-year period were used. The standard rules set out in the reference texts of professional ethics and nursing rules were considered in the process of preparing the questionnaire. The cognitive level in designing questions was considered to be at knowledge, understanding and application levels.

Some of the questions were: “Getting oral consent from the patient, especially in the presence of the witness has the same value as the written consent?”, “If the patient refuses treatment and other nursing care, is it just enough to mention it in the nursing report?”, “Is recording nursing interventions before they are committed an offense?”, “When the patient asks the nurse to comment on his treatment, does the nurse have the right to refuse to do so?”, “Is it a nurse’s duty to get pre-operative consent?” and “Does the patient have the right to have a trusted person at diagnostic stages, including examinations?”

Content validity analysis was used to determine the validity of the instrument. For this purpose, the questionnaire was distributed among 12 panels of experts at Kermanshah Universities of Medical Sciences. These individuals were experts in the field of ethics and professional rules. They were asked to review the questionnaire in terms of fluency, clarity and relevance. It was then modified based on their opinions. Test-retest reliability was used to examine the reliability of the instrument. In this regard, the questionnaire was distributed among 30 nurses, and after a two-week interval, they were asked to answer the questionnaire. Correlation coefficient of the pre-test and post-test scores was 0.87, which was acceptable. These nurses were not included in the study. The awareness questionnaire included yes-now questions, in which yes and no answers were scored 1 and zero, respectively. The range of score for each questionnaire was 0–37.

### Data collection

In order to collect the data, the researcher referred to the workplaces of the randomly selected samples. If the samples were willing to participate in the study, written informed consent was taken and then the questionnaires were distributed among them and collected in the same session. If a person was unwilling to participate in the study, he/she was replaced by the previous or next person listed on the list of nurses.

### Data analysis

The collected data were analyzed by Statistical Package for Social Sciences (SPSS)-Version 20 using descriptive (mean and frequency percentage) and inferential (Kolmogorov-Smirnov (KS), Mann-Whitney U, and Kruskal-Wallis) statistics. KS test was applied to determine the normality of the distribution of the samples’ awareness. The results of this test indicated an abnormal distribution of the mean awareness of the samples and therefore nonparametric tests were used to analyze the data. Mann-Whitney U test was used to compare the mean awareness of nurses based on bivariate qualitative variables (like sex and education) and Kruskal-Wallis test was used based on multivariate variables (like working ward and job rank) and ranking quantitative variables (like work experience). Significance level was set at < 0.05.

### Ethics

Ethical approval was obtained from the Ethical Review Committee of KUMS. The purpose of the study was explained to all participants. Concerning the anonymity and confidentiality, the samples were assured. Written informed consent was obtained from all participants.

## Results

The mean age of participants was 31.1 ± 6.4. Accordingly, 58.8% (*n* = 153) of the participants were female, 62.7% (*n* = 163) were married, 97.3% (*n* = 253) had BSc. degree and 86.1% (*n* = 224) were working as nurses. About half of the participants (50.4%, *n* = 128) worked at internal ward and 39.4% (*n* = 100) worked at surgical ward. Their mean work experience was 8.6 ± 6.5 years. About half of the participants (49%, n = 128) attended the retraining courses on professional rules. It was also found that 18% (*n* = 46) of nurses had a history of patients’ complaints (Table 1).

**Table 1:** Demographic characteristics of nurses

Variables		N (%)
Sex	Male	107(41.2)
	female	153(58.8)
Marital status	single	97(37.3)
	married	163(62.7)
Job position	supervisor	6(2.3)
	Staff nurse	21(8.1)
	Head Nurse	9(3.5)
	Nurse	224(86.1)
Job experience(Yrs)	1–5	110(43)
	6–10	68(26.6)
	≥11	82(32)
Degree	BSc.	253(97.3)
	MSc.	7(2.7)
Ward	Medical	128(50.4)
	Surgical	106(41.7)
	Intensive care	26(10.2)
Type of employment	formal	66(25)
	contractual	194(75)
Participation in retraining courses on professional rules	Yes	49(121)
	No	132(51)

The nurses’ mean awareness of professional rules was 28.3 ± 4.0 out of 37. Supervisors and staff nurses also had the highest and lowest mean awareness scores (31.2 ± 0.5 vs. 27.3 ± 7.2, respectively). There was no statistically significant difference between them.

Nurses working at intensive care unit (ICU) and surgical wards had the highest and lowest mean awareness (30.2 ± 2.4 vs. 27.1 ± 5, respectively), and this difference was statistically significant (*p* < 0.001).

Mean awareness of nurses with MSc. degree was higher than those with BSc. degree (28.2 ± 4.0 vs. 27.7 ± 3.5, respectively), indicating no statistically significant difference.

Regarding age variable, the highest and lowest mean scores of awareness were reported for those less than 30 and older than 31 years (28.5 ± 3.5 vs. 27.9 ± 4.6, respectively). There was no statistically significant difference between these two age groups.

The mean awareness score of male nurses was higher than that of the female nurses (28.8 ± 4.9 vs. 28.6 ± 3.3, respectively). However, the difference was not statistically significant.

The highest and lowest mean awareness scores were found for the nurses with a working experience of 1–5 years and more than 11 years (28.6 ± 3.5 vs. 27.7 ± 4.8, respectively). However, the difference was not statistically significant.

The mean awareness score of nurses who participated in retraining courses on professional rules was higher than those who did not take the course (28.5 ± 3.5 vs. 28 ± 4.5, respectively). The difference was not statistically significant, though (Table 2).

**Table 2:** Comparison of mean knowledge of professional rules in terms of demographic variables

Variables		Mean ± Sd	*P*- value
Sex	Male	28.78 ± 4.91	Z = −0.726 *P* = 0.468
	Female	28.59 ± 3.26	
Marital status	Single	28.53 ± 3.76	Z = −0.640 *P* = 0.522
	Married	28.16 ± 4.2	
age	≤30	28.52 ± 3.54	Z = −0.528 *P* = 0. 598
	≥31	27.9 ± 4.61	
Job position	Supervisor	31.25 ± 0.5	X^2^ = 4.707 *P* = 0.195
	Staff nurse	27.33 ± 7.19	
	Head Nurse	30.11 ± 2.96	
	Nurse	28.29 ± 3.69	
Job experience(Yrs)	1–5	28.65 ± 3.55	X^2^ = 1.632 *P* = 0.442
	6–10	28.15 ± 3.81	
	≥11	27.76 ± 4.84	
Degree	BSc.	27.66 ± 3.51	Z = −0.354 *P* = 0. 723
	MSc.	28.25 ± 4.06	
Ward	Medical	28.76 ± 3.28	X^2^ = 13.685 *P* = 0.001*
	Surgical	27.07 ± 4.9	
	Intensive care	30.16 ± 2.4	
Participation in retraining courses on professional rules	Yes	28.5 ± 3.51	Z = − 0.830 *P = 0.*407
	No	28 ± 4.52	

## Discussion

In this study, 18% of the nurses had a history of complaints on the part of the patients. International reports indicate an ascending trend of complaints from health professional, especially nurses in various countries, which has resulted in various legal problems [9–11]. Lack of awareness of legal issues and professional rules among medical practitioners is one of the main reasons for the increased number of complaints [13]. The results of the study by Gündoğmuş et al. in Turkey showed 1015 nursing and midwifery lawsuits were reported to the Higher Health Council during 5 years, 21 of which were related to nursing and resulted in conviction of nine nurses [20]. Other statistics showed that reported complaints regarding the quality of healthcare in Sweden increased from about 22,500 complaints in 2002 to about 25,000 complaints in 2006 [14]. Therefore, nurses as the largest group of healthcare workers should have sufficient and up-to-date knowledge about professional rules so that they will be able to act professionally in different situations and will not be subjected to legal complications.

In this study, the nurses’ awareness of professional rules was not at a satisfactory level. Previous studies have indicated that most of nurses do not have enough knowledge about professional rules. The results of a study by Eilts-Köchling et al. showed that only 25% of German nurses were aware of codes of ethics [19]. The findings of a study showed that 47.6% (*n* = 107) of Iranian nurses had low and 52.4% (*n* = 118) had average awareness [17].

In a study by Hariharan et al., 20% of nurses had little knowledge about the professional rules and 34% of them had no knowledge of Nursing Codes. Furthermore, only 10% of nurses were aware of the content of the Helsinki Declaration and The Nuremberg Code [18]. The results of our study were in line with the findings of these studies and indicated undesirability of nurses’ knowledge of professional rules.

In Iran in 2001, the Patients’ Bill of Rights was prepared by the Ministry of Health and Medical Education (MOHME) for the first time and was communicated to all hospitals in the country [21]. The National Code of Ethics for Nurses has also been prepared under the supervision of the MOHME and has been communicated to all hospitals throughout the country [22]. On the other hand, in recent years professional nursing ethics course has been added to the curriculum of graduate nursing. Although these measures are valuable steps in the pursuit of patients’ rights, evidence suggests that some nurses still do not have sufficient knowledge about the provisions of this charter and should take appropriate measures in this regard.

In our study, no statistically significant difference among various nursing ranks with regard to professional awareness. In our opinion, all nursing ranks must be aware of the professional rules, act ethically and consider nursing codes as professionals.

We found that nurses working at Intensive Care Units and surgical wards had the highest and lowest mean scores of awareness, respectively. This difference was statistically significant, though. Due to the complex needs of critically ill patients, critical care nurses must have sufficient clinical competencies to handle safe care and make correct decisions in different emergency situations [23, 24].

In our opinion, all nurses are required to increase their knowledge about the professional rules. In the meantime, nurses working in intensive care unit, owing to the special physical and mental conditions of patients, have to pay enough attention to this issue because they may face new situations every moment, which requires immediate decision making. Hence, lack of knowledge about professional rules may subject them to lawsuits by patients.

In our study, no significant relationship was found between the nurses’ mean awareness of professional rules and their educational level. It is expected that increase in education lead to higher awareness of professional rules among nurses so that they can be a guide for other nurses. One of the possible reasons for the same level of awareness among nurses with MSc. degree and those with BSc. degree can be lack of a course on nursing codes and professional rules in their curriculum. However, this course has been recently added to the higher education curriculum. It is expected that inclusion of this course in the curriculum promote the knowledge of nurses about professional rules.

We did not find a significant relationship between the nurses’ mean awareness and their age. Adib-Hajbaghery and Azizi also found no significant relationship in this regard [17]. Although no significant relationship was found between age and nurses’ awareness, it is expected that the necessity and importance of awareness of professional rules becomes more apparent for nurses as they get older, so they make more effort to increase their professional knowledge in this regard.

In our study, no significant relationship was found between nurses’ awareness and their gender. Adib-Hajbaghery and Azizi also reported similar results [17]. We believe that all nurses, whether male or female, have to have enough knowledge about professional rules and try to meet the needs of patients by improving their professional competence, which in turn prevents many legal complications.

We found no significant relationship between the nurses’ mean awareness and their work experience. Adib-Hajbaghery and Azizi also reported similar findings [17]. Although, there was not a significant relationship between awareness of professional rules and work experience, based on their personal experience, the authors concluded that increased work experience would result in increased awareness of professional rules. We believe that senior nurses should act as a role model for novice nurses and can be a good guide for these nurses while strengthening their legal knowledge.

The results of our study showed no significant relationship between the nurses’ mean awareness and participation in retraining courses on professional rules. This can question the effectiveness of these courses. In recent years, nursing managers in Iran have paid special attention to the empowerment of nurses in various professional fields, particularly in professional rules and ethics, and have held various retraining courses in this regard. Further, it seems necessary to revise how to hold these courses, which are mostly presented face to face. So, it is suggested to apply new methods of training such as web-based approach, the effectiveness of which in increasing the nurses’ awareness has been confirmed by previous studies [25, 26].

In this study, data were collected through self-report, which might have affected the accuracy of data. The study was conducted in state hospitals affiliated with KUMS. It is recommended to conduct similar studies on nurses working at private hospitals and compare the findings.

## Conclusion

Nurses as the largest group of healthcare workers are responsible for the safety and health of patients, so they are expected to have professional behavior in clinical cases. For this purpose, they have to have full competence and mastery of their professional rules. Participants of this study did not have satisfactory knowledge about professional rules. Since it is necessary for nurses to be aware of professional rules to provide safe healthcare, it is suggested that senior nursing managers take necessary interventional measures to improve the nurses’ awareness and hold retraining courses based on new teaching methods.

## Abbreviations

BSc: Bachelor of Science
CCU: Critical Care Unit
ICU: Intensive Care Unit
KS: Kolmogorov-Smirnov
KUMS: Kermanshah University of Medical Sciences
MOHME: Ministry of Health and Medical Education
MSc: Master of Science.
SPSS: Statistical Package for Social Sciences

## References

[1] Mirzaei M (2013). Prevalence, types of medication errors and barriers to reporting errors by nurses in an educational Hospital in Kermanshah. Hayat.

[2] Mohammad Nejad I (2010). Evaluation of medication error in nursing students in four educational hospitals in Tehran. Iranian Journal of Medical Ethics and History of Medicine.

[3] Wienke A. Errors and pitfalls: briefing and accusation of medical malpractice–the second victim. GMS Curr Top Otorhinolaryngol Head Neck Surg. 2013;12. 10.3205/cto000102 PMC3884545 24403978

[4] Kumar H (2013). Legal awareness and responsibilities of nursing staff in administration of patient care in a trust hospital. J Clin Diagn Res.

[5] Jolaee S (2009). Nursing medication errors and its relationship with work condition in Iran University of Medical Sciences. Iranian Journal of Medical Ethics and History of Medicine.

[6] Röing M, Holmström IK (2015). Malpractice claims in Swedish telenursing: lessons learned from interviews with telenurses and managers. Nurs Res.

[7] Röing M, Rosenqvist U, Holmström IK (2013). threats to patient safety in telenursing as revealed in Swedish telenurses' reflections on their dialogues. Scand J Caring Sci.

[8] Treiber LA, Jones JH (2010). Devastatingly human: an analysis of registered nurses’ medication error accounts. Qual Health Res.

[9] Gundogmus UN (2005). A descriptive study of medical malpractice cases in Turkey. Ann Saudi Med.

[10] Özdemir M, Ergönen T, Can I (2009). Medical malpractice claims involving children. Forensic Sci Int.

[11] Toraldo DM, Vergari U, Toraldo M (2015). Medical malpractice, defensive medicine and role of the “media” in Italy. Multidiscip Respir Med.

[12] Croke EM (2003). Nurses, negligence, and malpractice: an analysis based on more than 250 cases against nurses. AJN The American Journal of Nursing.

[13] Bagherian Mahmoud Abadi H (2014). Frequency and causes of obstetric negligence in referral cases to the General Office of Legal Medicine of Isfahan from 2005 to 2009. Iranian Journal of Obstetrics, Gynecology and Infertility.

[14] Jangland E, Gunningberg L, Carlsson M (2009). Patients’ and relatives’ complaints about encounters and communication in health care: evidence for quality improvement. Patient Educ Couns.

[15] Reising DL, Allen PN (2007). Protecting yourself from malpractice claims. American Nurse Today.

[16] Carson-Smith W, Klein C (2003). NP errors lead to litigation. Nurse Pract.

[17] Adib-Hajbaghery M, Azizi Fini E (2011). Nurses and nursing students’ knowledge of professional laws and regulations. Iranian Journal of Forensic Medicine(IJFM).

[18] Hariharan S (2006). Knowledge, attitudes and practice of healthcare ethics and law among doctors and nurses in Barbados. BMC Med Ethics.

[19] Eilts-Köchling K (2000). Knowledge of professional codes of ethics among nursing professionals. Pflege.

[20] Gündoğmuş ÜN, Özkara E, Mete S (2004). Nursing and midwifery malpractice in Turkey based on the higher health council records. Nurs Ethics.

[21] Mastaneh Z, Mouseli L (2013). Patients’ awareness of their rights: insight from a developing country. Int J Health Policy Manag.

[22] Zahedi F (2013). The code of ethics for nurses. Iran J Public Health.

[23] Deacon KS (2017). The National Competency Framework for registered nurses in adult critical care: an overview. J Intensive Care Soc.

[24] Mazaherpur S (2016). The Effect of Continuous Enteral Nutrition on Nutrition Indices, Compared to the Intermittent and Combination Enteral Nutrition in Traumatic Brain Injury Patients. J Clin Diagn Res.

[25] Khatony A (2009). The effectiveness of web-based and face-to-face continuing education methods on nurses' knowledge about AIDS: a comparative study. BMC Med Educ.

[26] Maloney S (2011). Effectiveness of web-based versus face-to-face delivery of education in prescription of falls-prevention exercise to health professionals: randomized trial. J Med Internet Res.

